# Potential Chemopreventive Role of Proton Pump Inhibitors in Head and Neck Cancer: Insights from a Nested Case–Control Analysis of a National Health Screening Cohort

**DOI:** 10.3390/jpm15010008

**Published:** 2024-12-28

**Authors:** Joong Seob Lee, Soomin Jo, Ho Suk Kang, Mi Jung Kwon, Jee Hye Wee, Jeong Wook Kang, Hyo Geun Choi, Heejin Kim

**Affiliations:** 1 Department of Otorhinolaryngology-Head & Neck Surgery, Hallym University Sacred Heart Hospital, Anyang 14068, Republic of Korea; apniosio@naver.com (J.S.L.); tnalswh07@gmail.com (S.J.); weejh07@gmail.com (J.H.W.); entkang@hallym.or.kr (J.W.K.); 2 Department of Internal Medicine, Hallym University Sacred Heart Hospital, Anyang 14068, Republic of Korea; hskang76@hallym.or.kr; 3 Department of Pathology, Hallym University Sacred Heart Hospital, Anyang 14068, Republic of Korea; mulank99@hallym.or.kr; 4 Suseo Seoul ENT Clinics, Seoul 05355, Republic of Korea; pupen@naver.com; 5 Md Analytics, Seoul 05355, Republic of Korea

**Keywords:** head and neck cancer, proton pump inhibitor, cohort study

## Abstract

**Background/Objectives**: This study investigated the potential chemopreventive role of proton pump inhibitor (PPI) use in relation to the occurrence of head and neck cancer (HNC) within a national cohort amid concerns of PPI overprescription. **Methods**: From a cohort of 1,137,861 individuals and 219,673,817 medical claim records collected between 2005 and 2019, 1677 HNC patients were identified and matched 1:4 with 6708 controls after adjusting for covariates. Odds ratios (ORs) for PPI use and its duration in relation to HNC and its subsites were estimated using propensity score overlap-weighted multivariable logistic regression. Additional subgroup analyses were performed based on age, sex, income level, and geographic region. **Results**: In the crude model, both current (OR 7.85 [95% CI 6.52–9.44]) and past PPI (OR 1.44 [95% CI 1.23–1.70]) use were associated with increased odds for HNC. However, after overlap weighting, this association reversed for both current (aOR 0.14 [95% CI 0.11–0.17]) and past PPI (aOR 0.69 [95% CI 0.60–0.79]). Subsite analysis showed reduced odds for hypopharyngeal (aOR 0.33, [95% CI 0.25–0.43]) and laryngeal cancer (aOR 0.19 [95% CI 0.16–0.22]) in current PPI users and similar results for past users. **Conclusions**: This study suggests a potential chemopreventive effect of PPIs, particularly in hypopharyngeal and laryngeal cancers. Additional studies are required to investigate the mechanisms underlying the association of the development of HNC with PPI use.

## 1. Introduction

Proton pump inhibitors (PPIs) are some of the most widely used medications worldwide. Otorhinolaryngologists often prescribe this medication to address persistent throat issues, including globus sensation (feeling of a lump in the throat), voice changes, and frequent throat clearing. A cohort study conducted in China [1] reported that 21.46% of respondents had experienced globus at some point in their lifetime. Many otorhinolaryngologists believe that globus symptoms are linked to esophageal conditions, especially laryngopharyngeal reflux (LPR). LPR is defined as the retrograde flow of gastric contents into the larynx and pharynx via the esophagus [2]. Unlike the esophageal mucosa, which is more resistant to gastric acid, the larynx and pharynx are highly sensitive, making patients with LPR more prone to experiencing laryngeal symptoms. However, the exact etiology of globus remains unclear, and there is no standardized investigation strategy. As demonstrated in a global study on LPR [3], PPIs are commonly prescribed once or twice a day for LPR treatment, with many clinicians recommending treatment for 2 to 3 months.

PPIs are potent inhibitors of gastric acid secretion, functioning by irreversibly binding to and blocking the hydrogen-potassium ATPase pump located on the luminal surface of parietal cell membranes. Although PPIs are considered to have minimal adverse effects, concerns about their overprescription have emerged in various studies [4]. As PPI usage increases, reports of side effects, particularly with long-term use, are also rising [5]. These side effects include increased susceptibility to infections, secondary hypergastrinemia, impaired micronutrient absorption, and idiosyncratic reactions. Our group previously reported an association between esophageal cancer and PPI use in a nationwide cohort study [6]. Additionally, numerous studies have examined the relationship between PPI use and the risk of gastric or colorectal cancer. A recent meta-analysis found that PPI use significantly increased the risk of gastric and colorectal cancer in Asian populations [7]. The head and neck region, like the esophagus, is lined with squamous cell epithelium. However, studies exploring the potential link between PPI use and head and neck cancer (HNC) are limited.

Therefore, this study aimed to explore the potential relationship between PPI use and the occurrence of HNC through an analysis of a national cohort.

## 2. Materials and Methods

### 2.1. Ethics

This study was approved by the Hallym University Ethics Committee (2022-10-008). The Institutional Review Board waived the requirement for written informed consent. All procedures were conducted in accordance with the ethical guidelines and regulations of the ethics committee of Hallym University.

### 2.2. Exposure (Proton Pump Inhibitor)

PPI use was defined by prescription and duration within the year preceding the index date [8]. PPI users were categorized into two groups: (1) those who used a PPI and (2) those with a specific duration of PPI use.

(1) The participants were categorized into three groups: nonusers, current PPI users (prescribed at least once within the past 30 days), and PPI-exposed individuals (prescribed at least once within 31–365 days).
(2) Participants were further divided into four groups based on the duration of PPI use: nonusers, 1–29 days of PPI use, 30–89 days of PPI use, and 90 or more days of PPI use.

### 2.3. Outcome (Head and Neck Cancer (HNC))

HNC was defined using the ICD-10 codes C00-C06 (oral cavity cancer), C09-10 (oropharynx cancer), C11 (nasopharynx cancer), C12-13 (hypopharynx cancer), C07-C08 (salivary gland cancer), C30(Not C301)-C31 (nasal cavity/sinus cancer), and C14. From this group, we identified participants with specific cancer-related claim codes (V193 and V194).

### 2.4. Participant Selection

A detailed description of the Korean National Health Insurance Service–National Sample Cohort (NHIS-NSC, from 2002 to 2019) is provided elsewhere [9].

HNC cases were identified from a cohort of 1,137,861 individuals with 219,673,817 medical claim records spanning 2005 to 2019 (*n* = 1677). The control group included participants who were not diagnosed with HNC between 2005 and 2019 (*n* = 1,136,184), with exclusions made for 3832 control participants who were diagnosed with HNC at least once. HNC cases were matched 1:4 with controls based on age, sex, income, and region of residence. To reduce selection bias, controls were randomly selected. The index date for each person with HNC was defined as the first treatment date for HNC, while the index date the for controls corresponded to the index date of their matched HNC participant.

**Figure 1:**
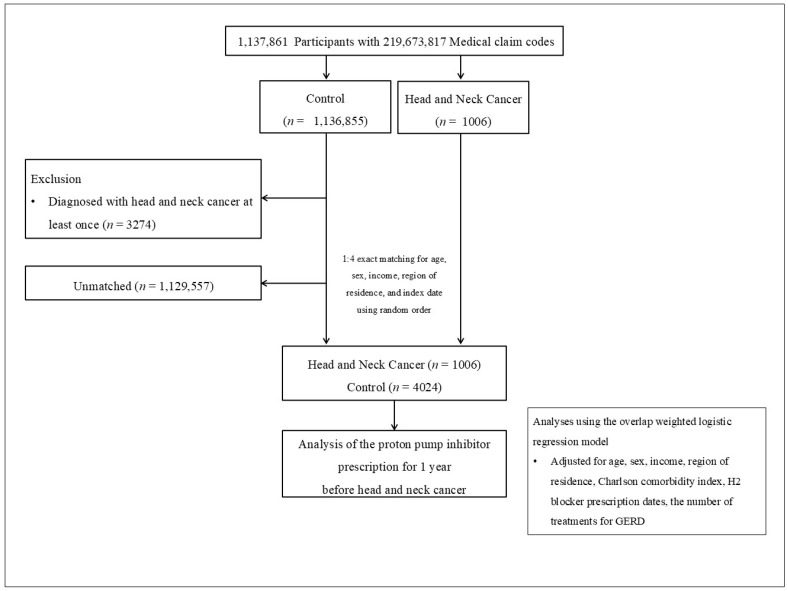
A flowchart depicting the participant selection process in this study. Out of 1,136,184 participants, 1677 individuals with head and neck cancer were matched to 6708 control participants based on age, sex, income, and geographic region.

Consequently, each matched HNC participant and their corresponding control had the same index date. During the matching process, 1,125,644 of the control participants were excluded. In the end, 1677 people with HNC were successfully matched 1:4 with 6708 control participants (Figure 1).

### 2.5. Covariates

Age was categorized into 5-year intervals: 0–4, 5–9, 10–14, and so on, up to 85+ years (for a total of 18 age groups). Income was stratified into five levels (Class 1 being the lowest income and Class 5 the highest) [10]. Residential area was classified into urban and rural regions based on the criteria from our previous study [11].

The Charlson comorbidity index (CCI), which is commonly used to assess disease burden based on 17 comorbidities, was applied. A score was assigned to each participant based on the severity and number of comorbid conditions. The CCI was treated as a continuous variable, ranging from 0 (no comorbidities) to 29 (multiple comorbidities) [12,13]. For this study, cancer was excluded from the CCI score calculation.

The frequency of treatment for gastro-esophageal reflux disease (GERD, ICD-10 code: K21), defined as receiving treatment at least twice and being prescribed a PPI for a minimum of 2 weeks, was evaluated for the year preceding the index date.

Additionally, the total number of H2 blocker prescriptions within 365 days before the index date was calculated.

### 2.6. Statistical Analyses

We applied propensity score overlap weighting to account for covariate balance and optimize the effective sample size. The propensity score (PS) was derived using multivariable logistic regression, including all covariates. For overlap weighting, cases were assigned weights based on 1-PS, while control participants were weighed by PS. This method produces overlap weights ranging from 0 to 1, achieving exact balance and improving precision [14,15,16].

The standardized difference was utilized to assess the differences in the general characteristics between the HNC and the control group (Table 1).

To evaluate the overlap-weighted odds ratios (ORs) for PPI use and its duration, the analysis focused on HNC, as well as specific subtypes including oral cavity, oropharynx, nasopharynx, hypopharynx, salivary gland, nasal cavity/sinus, and larynx cancers.

Propensity score overlap-weighted multivariable logistic regression was performed. Both crude (unadjusted) and overlap-weighted models (adjusted for age, sex, income, region of residence, CCI, H2 blocker prescription dates, and number of GERD treatments) were employed. In addition, subgroup analyses based on age, sex, income, and region of residence were conducted (Table 1).

The 95% confidence interval (CI) was computed, and two-tailed tests were used, with significance set at *p* values of less than 0.05. Statistical analyses were carried out using SAS version 9.4 (SAS Institute Inc., Cary, NC, USA).

## 3. Results

### 3.1. General Characteristics

In this study, 1677 patients with HNC and 6708 individuals in a comparison group were registered after PS matching. Table 1 presents the baseline characteristics of both groups before and after PS adjustment using the overlap weighting method. Patients with HNC were found to have a greater number of comorbidities, more frequent episodes of GERD, and a higher cumulative use of PPIs or H2 blockers compared to the control participants. Specifically, 67.68% (1135/1677) of those with HNC and 85.48% (2864/6708) of the control participants were non-PPI users.

Following the adjustment for group imbalances through overlap weighting, the standardized mean differences were reduced to zero, indicating that the baseline covariates were balanced (Table 1).

### 3.2. Associations of Prior Use of PPI and Its Duration with HNC

We examined the potential association between PPI use and the incidence of HNC in comparison to a control group (Table 2). In the crude model, the current PPI users exhibited significantly higher odds for developing HNC compared to the nonusers (odds ratio [OR] 7.85 [95% CI 6.52–9.44, *p* < 0.001]). However, this association reversed in the adjusted model using overlap weighting (adjusted odds ratio [aOR] with overlap weighting [OW] 0.14 [95% CI 0.11–0.17; *p* < 0.001]). Similarly, a history of PPI exposure was initially associated with increased odds of HNC in the crude model (OR 1.44 [95% CI 1.23–1.70, *p* < 0.001]), but this association reversed after adjustment with overlap weighting ([aOR] with OW 0.69 [95% CI 0.60–0.79; *p* < 0.001]).

**Figure 2:**
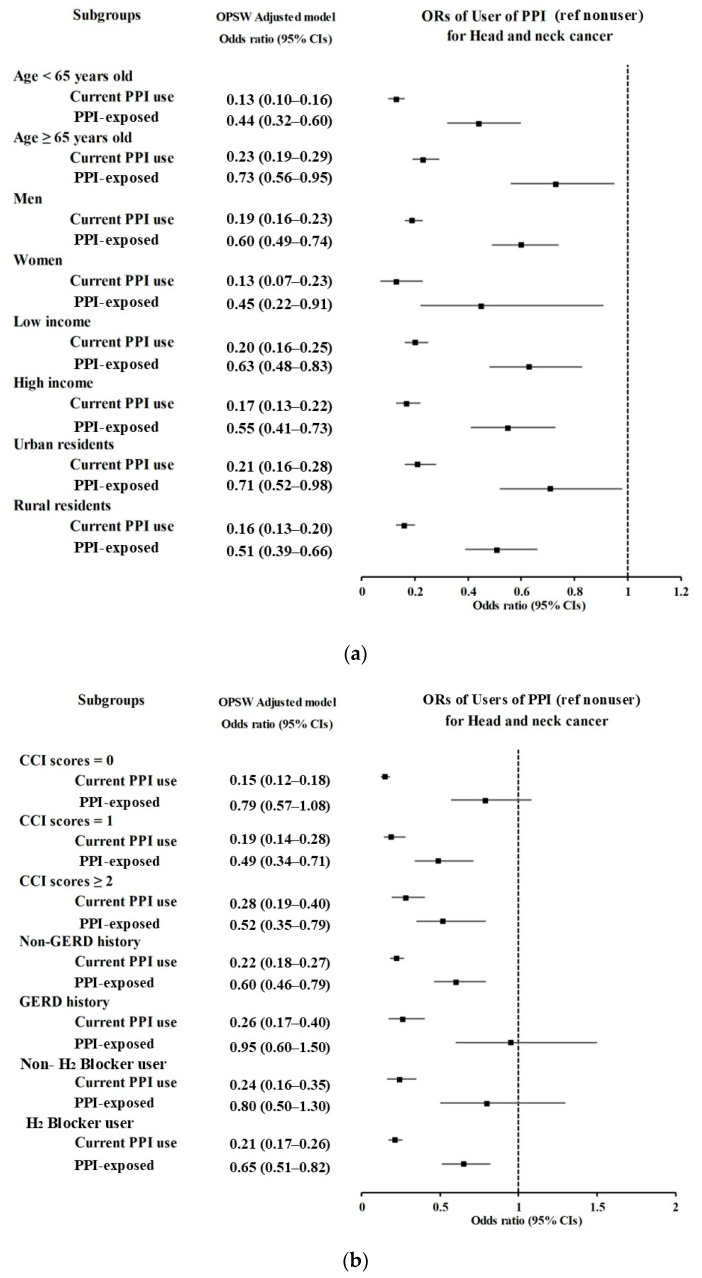
Subgroup analyses of proton pump inhibitor (PPI) use (nonuser [ref] vs. user) for head and neck cancer (HNC) based on age, sex, income, and region of residence presented in a forest plot (**a**). (**b**) Subgroup analyses of PPI use for HNC according to comorbidities (Charlson comorbidity index [CCI]), history of gastroesophageal reflux disease (GERD), and H_2_ blocker use), also visualized in a forest plot.

PPI use was linked to decreased odds of HNC, particularly among individuals younger than 65 years, women, and those without a history of GERD, as illustrated in the forest plots (Figure 2a,b) and Table S1. The likelihood of developing HNC decreased with the length of PPI use, with adjusted odds ratios of 0.30 [95% CI 0.26–0.35, *p* < 0.001] for less than 30 days, 0.44 [95% CI 0.36–0.53, *p* < 0.001] for 30–89 days, and 0.61 [95% CI 0.49–0.77, *p* < 0.001] for over 90 days (Table 3).

**Figure 3:**
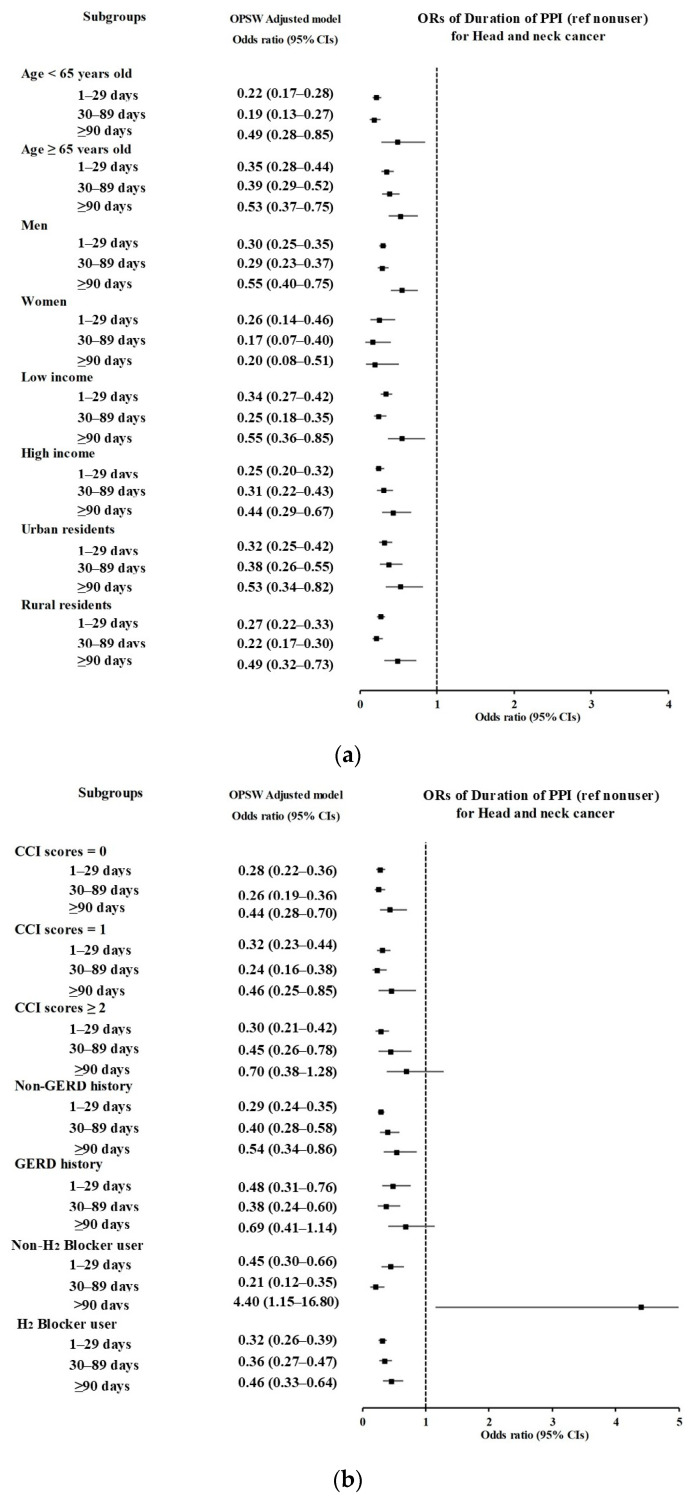
Subgroup analyses of the duration of proton pump inhibitor (PPI) use (<30 day [ref] versus ≥30 days) for head and neck cancer (HNC) according to age, sex, income, and region of residence (**a**), as well as comorbidities (Charlson comorbidity index [CCI]), history of gastroesophageal reflux disease (GERD), and H_2_ blocker use (**b**), visualized as a forest plot.

In the subgroup analyses, those with PPI use for less than 90 days consistently showed a lower probability of developing HNC across various factors, including age, sex, income, residence, and number of comorbidities (Figure 3a,b, and Table S2). Notably, the subgroup using non-H2 blockers showed an association between PPI use for 90 days or more and HNC risk (aOR 4.40 [95% CI 1.15–16.8, *p* < 0.031], Table S2).

### 3.3. Associations Between the Use of PPI and HNC According to Subsites

The head and neck region encompasses various subsites. When analyzing people with HNC by subsite, Table 2 shows that current PPI users had a lower likelihood of developing oropharynx cancer (aOR with OW 0.70 [95% CI 0.53–0.94, *p* = 0.016]), nasopharynx cancer (aOR with OW 0.68 [95% CI 0.49–0.94, *p* = 0.018]), hypopharynx cancer (aOR with OW 0.33, [95% CI 0.25–0.43, *p* < 0.001]), salivary gland cancer (aOR with OW 0.42, [95% CI 0.32–0.56, *p* < 0.001]), and larynx cancer (aOR with OW 0.19, [95% CI 0.16–0.22, *p* < 0.001]) compared to nonusers. Additionally, a history of PPI use was associated with a reduced likelihood of hypopharynx cancer (aOR with OW 0.71, [95% CI 0.51–0.99, *p* = 0.042]) and larynx cancer (aOR with OW 0.59, [95% CI 0.48–0.72, *p* < 0.001]).

The duration of PPI use significantly impacted the risk of developing hypopharyngeal and laryngeal cancers. For hypopharyngeal cancer, the aORs with OW for different durations of PPI use were as follows: 1–29 days (0.44, [95% CI 0.34–0.57, *p* < 0.001]), 30–89 days (0.54, [95% CI 0.36–0.81, *p* = 0.003]), and over 90 days (0.43, [95% CI 0.27–0.67, *p* < 0.001]) (Table 3). Similarly, for laryngeal cancer, the aORs with OW were 1–29 days (0.30, [95% CI 0.25–0.35, *p* < 0.001]), 30–89 days (0.28, [95% CI 0.23–0.36, *p* < 0.001]), and over 90 days (0.50, [95% CI 0.37–0.68, *p* < 0.001]) (Table 3).

Interestingly, salivary gland cancer was positively associated with prior PPI use (aOR with OW 1.57, [95% CI 1.05–2.36, *p* = 0.027]), which contrasts the association observed with current PPI use. Additionally, the risk of developing salivary gland cancer did not appear to be influenced by the duration of PPI use.

## 4. Discussion

In this national cohort study, we identified an association between PPI use and the occurrence of HNC, particularly in hypopharyngeal and laryngeal cancers. Interestingly, while the crude model initially demonstrated a positive relationship between PPI use and HNC occurrence, this association changed after adjusting for covariates using adjusted odds ratios (aORs) with overlap weighting (OW). Many patients with HNC reported throat symptoms and frequently used medications such as PPIs, which may have led to the misconception that PPI use was related to the occurrence of HNC. However, our study revealed a chemopreventive effect of PPIs, particularly in hypopharyngeal and laryngeal cancers.

The well-established theory linking acid reflux to HNC has particularly emphasized laryngeal cancer. A meta-analysis on GERD and the subsequent diagnosis of HNC revealed a significant association with laryngeal cancer, but not with other regions, such as the oropharynx and hypopharynx [17]. Nonetheless, the authors highlighted a potential significant clinical association with the development of pharyngeal cancers, particularly when evaluating the entire confidence interval. The authors proposed that GERD symptoms might trigger a metaplasia–dysplasia–carcinoma sequence, similar to Barret’s esophagus, suggesting that a comparable mechanism could occur in laryngeal tissues. Our group also previously identified an association between GERD and laryngeal cancer using data from a national sample cohort, but no such link for other subsites, including the oral cavity and pharyngeal cancer [18]. Additionally, a study using electron microscopy and qPCR on laryngeal tissues demonstrated the expression of gastric proton pump subunits in the larynx of patients with laryngeal squamous cell carcinoma [19]. These findings have emphasized the potential chemo-preventive role of antacid medications in HNC pathobiology. Papagerakis et al. also confirmed that patients diagnosed with HNC benefit from the use of antacid medications (H2RA and PPI) in their study [20].

A previous meta-analysis revealed that the association between GERD and HNC may be specific to laryngeal cancer, rather than hypopharyngeal cancer [16]. However, our results also indicate that PPI use may be beneficial in reducing the occurrence of hypopharyngeal cancer. Unlike the esophageal mucosa, the laryngeal and hypopharyngeal mucosa lacks carbonic anhydrase, the enzyme responsible for producing bicarbonate [21]. As a result, the laryngopharyngeal mucosa has limited self-protection and is particularly vulnerable to damage from LPR stimulation [22,23,24]. The diagnosis of LPR remains controversial due to the absence of a gold standard; some studies rely on esophagoscopy, clinical symptoms, questionnaires, or 24-h dual-probe pH measurement, among others. One study identified pepsin as a marker for diagnosing LPR, and demonstrated experimentally that pepsin can caused epithelial damage in the hypopharyngeal mucosa [25]. Other research has indicated a potential link between bile reflux and hypopharyngeal carcinogenesis [26,27]. PPIs may play a role in reducing bile reflux, as one study reported that PPIs significantly shortened the duration of bile reflux in GERD patients, though they did not always normalize it [28]. Numerous studies have also proposed a link between *H. pylori* and laryngopharyngeal cancer [29,30]. Additionally, PPI use may be beneficial for eradicating *H. pylori* in combination with antibiotics as part of triple or quadruple therapy [31].

PPIs are widely used, and their use has increased in many countries due to the perception that they have few adverse events and are generally well tolerated. However, concerns about overprescription have arisen, particularly regarding long-term use. Several studies have investigated the relationship between prolonged PPI use and all-cause mortality, with a focus on the potential link between PPIs and cancer. The association between PPI exposure and gastric cancer has been the subject of extensive debate regarding adverse health outcomes. However, a recent meta-analysis study found no significant association between PPI use and gastric cancer [32]. Unlike the case of gastric cancer, several studies have demonstrated a chemopreventive effect of PPIs on esophageal cancer [33,34].

This study has several notable strengths. It is based on a nationally representative cohort database with well-matched patient and control groups, enhancing the generalizability of our findings. The Korean National Health Insurance Service-Health Screening Cohort (KNHIS-HSC) data used in this study include records from all hospitals and clinics nationwide, ensuring their representative value. Additionally, this study initially found a positive association between PPI use and HNC occurrence in the crude model, suggesting that the observed overuse of PPIs could have been misconceived as contributing to HNC risk. However, after adjusting for confounding factors, we detected a chemopreventive effect of PPIs on HNC, a unique finding that has rarely been reported in previous studies.

This study has several limitations that should be acknowledged. First, the health insurance dataset lacked information on critical factors such as *H. pylori* infection, histological data, family history, and genetic information related to HNC, which may have led to missing or incomplete data. Furthermore, our evaluation did not account for lifestyle confounding factors, including well-known carcinogens such as smoking and alcohol use, which are important in understanding the risk of HNC. Second, patient adherence to medication could not be assessed through the KNHIS-HSC data. Third, as this study relied on diagnosis codes and focused solely on Korean participants, unmeasured confounding variables could not be entirely ruled out. Differences related to race, diet, environmental factors, and other variables may exist.

## 5. Conclusions

This study carefully explored the potential association between PPI use and the incidence of HNC in the Korean population. We found a significant link between PPI use and the occurrence of hypopharyngeal and laryngeal cancers. Interestingly, while the crude model initially showed a positive relationship between PPI use and HNC, this relationship shifted after adjusting for covariates using aOR with OW. Our findings suggest a potential chemopreventive effect of PPIs, particularly in hypopharyngeal and laryngeal cancers. Further studies are needed to elucidate the underlying pathophysiologic mechanisms involved in HNC development.

## Figures and Tables

**Table 1:** General characteristics of participants.

Characteristics	Before ^2^ PS Overlap Weighting Adjustment			After PS Overlap Weighting Adjustment		
	Head and Neck Cancer	Control	Standardized Difference	Head and Neck Cancer	Control	Standardized Difference
Total participants (*n*, %)	1677 (100)	6708 (100)		1324 (100)	1324 (100)	
Age (%)			0.00			0.00
Under 40	99 (5.90)	396 (5.90)		79 (5.92)	79 (5.92)	
40–44	65 (3.88)	260 (3.88)		51 (3.88)	51 (3.88)	
45–49	112 (6.68)	448 (6.68)		88 (6.67)	88 (6.67)	
50–54	178 (10.61)	712 (10.61)		140 (10.56)	140 (10.56)	
55–59	218 (13.00)	872 (13.00)		173 (13.06)	173 (13.06)	
60–64	225 (13.42)	900 (13.42)		176 (13.31)	176 (13.31)	
65–69	220 (13.12)	880 (13.12)		174 (13.10)	174 (13.10)	
70–74	233 (13.89)	932 (13.89)		183 (13.80)	183 (13.80)	
75–79	173 (10.32)	692 (10.32)		137 (10.35)	137 (10.35)	
80–84	102 (6.08)	408 (6.08)		82 (6.17)	82 (6.17)	
85+	52 (3.10)	208 (3.10)		42 (3.16)	42 (3.16)	
Sex (%)						
Male	1255 (74.84)	5020 (74.84)	0.00	988 (74.60)	988 (74.60)	0.00
Female	422 (25.16)	1688 (25.16)		336 (25.40)	336 (25.40)	
Income (%)						
1 (lowest)	370 (22.06)	1480 (22.06)	0.00	293 (22.09)	293 (22.09)	0.00
2	229 (13.66)	916 (13.66)		181 (13.65)	181 (13.65)	
3	248 (14.79)	992 (14.79)		196 (14.78)	196 (14.78)	
4	347 (20.69)	1388 (20.69)		274 (20.65)	274 (20.65)	
5 (highest)	483 (28.80)	1932 (28.80)		382 (28.83)	382 (28.83)	
Region of residence (%)						
Urban	716 (42.70)	2864 (42.70)	0.00	567 (42.82)	567 (42.82)	0.00
Rural	961 (57.30)	3844 (57.30)		757 (57.18)	757 (57.18)	
^1^ CCI score (mean, SD)	0.90 (1.23)	0.68 (1.18)	0.19	0.85 (1.05)	0.85 (0.61)	0.00
Number of treatments with ^3^ GERD (mean, SD)	0.79 (2.62)	0.41 (1.77)	0.17	0.65 (1.92)	0.65 (1.13)	0.00
H_2_ blocker prescription dates (*n*, %)	34.06 (69.49)	25.29 (64.86)	0.13	31.94 (58.82)	31.94 (33.61)	0.00
User of ^4^ PPI (*n*, %)			0.54			0.49
Nonuser	1135 (67.68)	5734 (85.48)		909 (68.64)	1104 (83.35)	
Current PPI use	323 (19.26)	208 (3.10)		248 (18.71)	53 (4.03)	
PPI-exposed	219 (13.06)	766 (11.42)		168 (12.65)	167 (12.61)	
Duration of PPI use (*n*, %)						
Nonuser	1135 (67.68)	5734 (85.48)	0.43	909 (68.64)	1104 (83.35)	0.39
1–29 days	323 (19.26)	474 (7.07)		253 (19.12)	99 (7.45)	
30–89 days	129 (7.69)	260 (3.88)		98 (7.39)	60 (4.51)	
≥90 days	90 (5.37)	240 (3.58)		64 (4.86)	62 (4.69)	

^1^ CCI, Charlson comorbidity index; ^2^ PS, propensity score; ^3^ GERD, gastroesophageal reflux disease; ^4^ PPI, proton pump inhibitor.

**Table 2:** Crude and overlap propensity score-weighted odd ratios for proton pump inhibitor (PPI) use (reference: nonusers) in relation to head and neck cancer, including specific subtypes: oral cavity cancer, oropharynx cancer, nasopharynx cancer, hypopharynx cancer, salivary gland cancer, nasal cavity/sinus cancer, and larynx cancer.

Characteristics	Number of Events (Exposure/Total, %)	Number of Controls (Exposure/Total, %)	Odds Ratios (95% Confidence Intervals)			
			Crude	*p*-Value	Adjusted Model with ^1^ OW †	*p*-Value
Odd ratios for head and neck cancer						
Current PPI use	323/1677 (19.26)	208/6708 (3.1)	7.85 (6.52–9.44)	<0.001 *	0.14 (0.11–0.17)	<0.001 *
^2^ PPI-exposed	219/1677 (13.06)	766/6708 (11.42)	1.44 (1.23–1.70)	<0.001 *	0.69 (0.60–0.79)	<0.001 *
Odd ratios for oral cavity cancer						
Current PPI use	41/409 (10.02)	490/7976 (6.14)	1.74 (1.24–2.44)	0.001 *	1.01 (0.80–1.27)	0.964
PPI-exposed	53/409 (12.96)	932/7976 (11.69)	1.18 (0.88–1.60)	0.27	0.89 (0.72–1.11)	0.302
Odd ratios for oropharynx cancer						
Current PPI use	27/195 (13.85)	504/8190 (6.15)	2.52 (1.65–3.84)	<0.001 *	0.70 (0.53–0.94)	0.016 *
PPI-exposed	25/195 (12.82)	960/8190 (11.72)	1.22 (0.80–1.88)	0.355	0.79 (0.59–1.07)	0.124
Odd ratios for nasopharynx cancer			183 (13.80)		183 (13.80)	
Current PPI use	21/167 (12.57)	510/8218 (6.21)	2.14 (1.34–3.42)	0.002 *	0.68 (0.49–0.94)	0.018 *
PPI-exposed	16/167 (9.58)	969/8218 (11.79)	0.86 (0.51–1.44)	0.56	1.06 (0.74–1.52)	0.743
Odd ratios for hypopharynx cancer						
Current PPI use	41/143 (28.67)	490/8242 (5.95)	7.01 (4.76–10.3)	<0.001 *	0.33 (0.25–0.43)	<0.001 *
PPI-exposed	21/143 (14.69)	964/8242 (11.7)	1.83 (1.12–2.96)	0.015 *	0.71 (0.51–0.99)	0.042 *
Odd ratios for salivary gland cancer						
Current PPI use	30/177 (16.95)	501/8208 (6.1)	2.99 (1.99–4.48)	<0.001 *	0.42 (0.32–0.56)	<0.001 *
PPI-exposed	12/177 (6.78)	973/8208 (11.85)	0.62 (0.34–1.11)	0.109	1.57 (1.05–2.36)	0.027 *
Odd ratios for nasal cavity/sinus cancer						
Current PPI use	9/131 (6.87)	522/8254 (6.32)	1.17 (0.59–2.32)	0.66	1.48 (0.93–2.36)	0.096
PPI-exposed	22/131 (16.79)	963/8254 (11.67)	1.55 (0.97–2.47)	0.067	0.72 (0.52–1.00)	0.049 *
Odd ratios for larynx cancer						
Current PPI use	150/444 (33.78)	381/7941 (4.8)	11.5 (9.11–14.4)	<0.001 *	0.19 (0.16–0.22)	<0.001 *
PPI-exposed	66/444 (14.86)	919/7941 (11.57)	2.09 (1.58–2.77)	<0.001 *	0.59 (0.48–0.72)	<0.001 *

^1^ OW, overlap weighting; ^2^ PPI, proton pump inhibitor. * Significance at *p* < 0.05. † Adjusted for age, sex, income, region of residence, CCI score, H2 blocker prescription dates, and the number of treatments for GERD.

**Table 3:** Crude and overlap propensity score-weighted odd ratios for duration of proton pump inhibitor (PPI) use (reference: nonusers) in relation to head and neck cancer, including subtypes: oral cavity cancer, oropharynx cancer, nasopharynx cancer, hypopharynx cancer, salivary gland cancer, nasal cavity/sinus cancer, and larynx cancer.

Characteristics	Number of Events (Exposure/Total, %)	Number of Controls (Exposure/Total, %)	Odds Ratios (95% Confidence Intervals)			
			Crude	*p*-Value	Adjusted Model with ^1^ OW †	*p*-Value
Odd ratios for head and neck cancer						
1–29 days	323/1677 (19.26)	474/6708 (7.07)	3.44 (2.95–4.02)	<0.001 *	0.30 (0.26–0.35)	<0.001 *
30–89 days	129/1677 (7.69)	260/6708 (3.88)	2.51 (2.01–3.12)	<0.001 *	0.44 (0.36–0.53)	<0.001 *
≥90 days	90/1677 (5.37)	240/6708 (3.58)	1.89 (1.47–2.43)	<0.001 *	0.61 (0.49–0.77)	<0.001 *
Odd ratios for oral cavity cancer						
1–29 days	63/409 (15.4)	734/7976 (9.2)	1.79 (1.35–2.37)	<0.001 *	0.81 (0.67–0.99)	0.035 *
30–89 days	16/409 (3.91)	373/7976 (4.68)	0.89 (0.53–1.49)	0.664	1.44 (1.01–2.05)	0.045 *
≥90 days	15/409 (3.67)	315/7976 (3.95)	0.99 (0.58–1.68)	0.973	1.12 (0.76–1.65)	0.573
Odd ratios for oropharynx cancer						
1–29 days	32/195 (16.41)	765/8190 (9.34)	1.97 (1.33–2.91)	<0.001 *	0.71 (0.55–0.92)	0.009 *
30–89 days	8/195 (4.1)	381/8190 (4.65)	0.99 (0.48–2.03)	0.973	1.18 (0.73–1.92)	0.491
≥90 days	12/195 (6.15)	318/8190 (3.88)	1.77 (0.97–3.23)	0.061	0.50 (0.31–0.78)	0.003 *
Odd ratios for nasopharynx cancer			183 (13.80)		183 (13.80)	
1–29 days	23/167 (13.77)	774/8218 (9.42)	1.54 (0.98–2.41)	0.06	0.82 (0.61–1.11)	0.196
30–89 days	10/167 (5.99)	379/8218 (4.61)	1.37 (0.71–2.62)	0.346	0.77 (0.49–1.22)	0.263
≥90 days	4/167 (2.4)	326/8218 (3.97)	0.64 (0.23–1.73)	0.376	1.30 (0.64–2.66)	0.472
Odd ratios for hypopharynx cancer						
1–29 days	35/143 (24.48)	762/8242 (9.25)	3.85 (2.57–5.76)	<0.001 *	0.44 (0.34–0.57)	<0.001 *
30–89 days	13/143 (9.09)	376/8242 (4.56)	2.90 (1.60–5.25)	<0.001 *	0.54 (0.36–0.81)	0.003 *
≥90 days	14/143 (9.79)	316/8242 (3.83)	3.71 (2.08–6.62)	<0.001 *	0.43 (0.27–0.67)	<0.001 *
Odd ratios for salivary gland cancer						
1–29 days	31/177 (17.51)	766/8208 (9.33)	2.02 (1.36–3.00)	<0.001 *	0.60 (0.46–0.79)	<0.001 *
30–89 days	8/177 (4.52)	381/8208 (4.64)	1.05 (0.51–2.15)	0.9	1.07 (0.64–1.77)	0.799
≥90 days	3/177 (1.69)	327/8208 (3.98)	0.46 (0.15–1.44)	0.183	2.19 (0.95–5.04)	0.064
Odd ratios for nasal cavity/sinus cancer						
1–29 days	14/131 (10.69)	783/8254 (9.49)	1.21 (0.69–2.13)	0.507	1.14 (0.79–1.65)	0.477
30–89 days	12/131 (9.16)	377/8254 (4.57)	2.16 (1.17–3.96)	0.013 *	0.57 (0.37–0.87)	0.01 *
≥90 days	5/131 (3.82)	325/8254 (3.94)	1.04 (0.42–2.57)	0.93	1.16 (0.60–2.24)	0.66
Odd ratios for larynx cancer						
1–29 days	120/444 (27.03)	677/7941 (8.53)	5.16 (4.08–6.53)	<0.001 *	0.30 (0.25–0.35)	<0.001 *
30–89 days	60/444 (13.51)	329/7941 (4.14)	5.31 (3.91–7.21)	<0.001 *	0.28 (0.23–0.36)	<0.001 *
≥90 days	36/444 (8.11)	294/7941 (3.7)	3.57 (2.46–5.17)	<0.001 *	0.50 (0.37–0.68)	<0.001 *

^1^ OW, overlap weighting. * Significance at *p* < 0.05. † Adjusted for age, sex, income, region of residence, CCI score, H2 blocker prescription dates, and the number of treatments for GERD.

## Data Availability

These data are subject to certain limitations regarding their availability. They were acquired from the Health Insurance Review and Assessment Service (HIRA) of Korea and can be accessed at https://opendata.hira.or.kr (accessed on 20 September 2023) with the authorization of the HIRA.

## Supplementary Materials

The following supporting information can be downloaded at: https://www.mdpi.com/article/10.3390/jpm15010008/s1, Table S1: Subgroup analyses of user of proton pump inhibitor for head and neck cancer; Table S2: Subgroup analyses regarding odds ratio of duration of proton pump inhibitor for head and neck cancer.
