# Supplementary material for: Potential Chemopreventive Role of Proton Pump Inhibitors in Head and Neck Cancer: Insights from a Nested Case–Control Analysis of a National Health Screening Cohort
Source: J Pers Med. 2024 Dec 28;15(1):8. doi: 10.3390/jpm15010008 (PMC11766546; doi:10.3390/jpm15010008)
Supplement: Supplementary file 1 [file jpm-15-00008-s001.zip › jpm-3323858-supplementary.pdf]

S1 Subgroup analyses regarding odds ratio (95% confidence intervals) of User of PPI (ref: non user) for Head and neck cancer

User of PPI	Head and neck cancer	Control	Odds ratios (95% confidence intervals)			
	(exposure/total, %)	(exposure/total, %)	Crude	P value	Adjusted model with OW†	P value
Age < 65 years old (*n* = 4485)						
Current PPI use	139/897 (15.5)	73/3588 (2.03)	16.6 (11.6-23.8)	<0.001*	0.13 (0.10-0.16)	<0.001*
PPI-exposed	103/897 (11.48)	344/3588 (9.59)	2.78 (1.81-4.28)	<0.001*	0.44 (0.32-0.60)	<0.001*
Age ≥ 65 years old (*n* = 3900)						
Current PPI use	184/780 (23.59)	135/3120 (4.33)	8.21 (6.08-11.1)	<0.001*	0.23 (0.19-0.29)	<0.001*
PPI-exposed	116/780 (14.87)	422/3120 (13.53)	1.59 (1.09-2.32)	0.015*	0.73 (0.56-0.95)	0.019*
Males (*n* = 6275)						
Current PPI use	277/1,255 (22.07)	166/5020 (3.31)	10.8 (8.46-13.7)	<0.001*	0.19 (0.16-0.23)	<0.001*
PPI-exposed	163/1,255 (12.99)	561/5020 (11.18)	2.12 (1.58-2.86)	<0.001*	0.60 (0.49-0.74)	<0.001*
Females (*n* = 2110)						
Current PPI use	46/422 (10.9)	42/1688 (2.49)	12.4 (5.33-29.0)	<0.001*	0.13 (0.07-0.23)	<0.001*
PPI-exposed	56/422 (13.27)	205/1688 (12.14)	2.13 (0.77-5.86)	0.143	0.45 (0.22-0.91)	0.026*
Low income (*n* = 4235)						
Current PPI use	166/847 (19.6)	106/3388 (3.13)	10.1 (7.36-13.9)	<0.001*	0.20 (0.16-0.25)	<0.001*
PPI-exposed	109/847 (12.87)	397/3388 (11.72)	1.90 (1.29-2.80)	0.001*	0.63 (0.48-0.83)	0.001*
High income (*n* = 4150)						

Current PPI use	157/830 (18.92)	102/3320 (3.07)	13.3 (9.47-18.6)	<0.001*	0.17 (0.13-0.22)	<0.001*
PPI-exposed	110/830 (13.25)	369/3320 (11.11)	2.34 (1.54-3.55)	<0.001*	0.55 (0.41-0.73)	<0.001*
Urban (*n* = 3,580)						
Current PPI use	140/716 (19.55)	87/2864 (3.04)	9.67 (6.79-13.8)	<0.001*	0.21 (0.16-0.28)	<0.001*
PPI-exposed	98/716 (13.69)	323/2864 (11.28)	1.70 (1.09-2.66)	0.02*	0.71 (0.52-0.98)	0.034*
Rural (*n* = 4,805)						
Current PPI use	183/961 (19.04)	121/3844 (3.15)	13.0 (9.62-17.7)	<0.001*	0.16 (0.13-0.20)	<0.001*
PPI-exposed	121/961 (12.59)	443/3844 (11.52)	2.43 (1.69-3.50)	<0.001*	0.51 (0.39-0.66)	<0.001*
CCI scores = 0 (*n* = 5,143)						
Current PPI use	144/850 (16.94)	95/4293 (2.21)	16.3 (11.6-23.0)	<0.001*	0.15 (0.12-0.18)	<0.001*
PPI-exposed	96/850 (11.29)	439/4293 (10.23)	1.76 (1.10-2.81)	0.018*	0.79 (0.57-1.08)	0.135
CCI scores = 1 (*n* = 1,754)						
Current PPI use	87/453 (19.21)	52/1301 (4)	9.52 (6.17-14.7)	<0.001*	0.19 (0.14-0.28)	<0.001*
PPI-exposed	68/453 (15.01)	169/1301 (12.99)	2.24 (1.37-3.65)	0.001*	0.49 (0.34-0.71)	<0.001*
CCI scores ≥ 2 (*n* = 1,488)						
Current PPI use	92/374 (24.6)	61/1114 (5.48)	5.85 (3.71-9.22)	<0.001*	0.28 (0.19-0.40)	<0.001*
PPI-exposed	55/374 (14.71)	158/1114 (14.18)	1.90 (1.12-3.23)	0.018*	0.52 (0.35-0.79)	0.002*
Non-GERD history (*n* = 7,326)						
Current PPI use	182/1,362 (13.36)	76/5964 (1.27)	10.9 (7.99-14.8)	<0.001*	0.22 (0.18-0.27)	<0.001*

PPI-exposed	101/1362 (7.42)	370/5964 (6.2)	1.92 (1.28-2.88)	0.002*	0.60 (0.46-0.79)	<0.001*
GERD history (*n* = 1059)						
Current PPI use	141/315 (44.76)	132/744 (17.74)	6.06 (3.52-10.4)	<0.001*	0.26 (0.17-0.40)	<0.001*
PPI-exposed	118/315 (37.46)	396/744 (53.23)	1.13 (0.63-2.01)	0.688	0.95 (0.60-1.50)	0.836
Non-H_2 blocker user (*n* = 3664)						
Current PPI use	46/432 (10.65)	45/3232 (1.39)	10.7 (5.93-19.4)	<0.001*	0.24 (0.16-0.35)	<0.001*
PPI-exposed	27/432 (6.25)	213/3232 (6.59)	1.51 (0.68-3.33)	0.307	0.80 (0.50-1.30)	0.375
H_2 blocker user (*n* = 4721)						
Current PPI use	277/1245 (22.25)	163/3476 (4.69)	9.06 (7.01-11.7)	<0.001*	0.21 (0.17-0.26)	<0.001*
PPI-exposed	192/1245 (15.42)	553/3476 (15.91)	1.78 (1.31-2.42)	<0.001*	0.65 (0.51-0.82)	<0.001*

Abbreviations: CCI, Charlson comorbidity index; GERD, gastro-esophageal reflux disease; OW, overlap weighting; PPI, proton pump inhibitor;

* Logistic regression model, Significance at P < 0.05

† Adjusted for age, sex, income, region of residence, CCI score, H2 blocker dates, and the number of GERD treatment

S2 Subgroup analyses regarding odds ratio (95% confidence intervals) of Duration of PPI (ref: non user) for head and neck cancer

Duration of PPI	Head and neck cancer	Control	Odds ratios (95% confidence intervals)			
	(exposure/total, %)	(exposure/total, %)	Crude	P value	Adjusted model with OW†	P value
Age < 65 years old (*n* = 4485)						
≥1 days & <30 days	157/897 (17.5)	233/3588 (6.49)	6.45 (4.53-9.19)	<0.001*	0.22 (0.17-0.28)	<0.001*
≥30 days & < 90 days	60/897 (6.69)	105/3588 (2.93)	7.85 (4.95-12.5)	<0.001*	0.19 (0.13-0.27)	<0.001*
≥ 90 days	25/897 (2.79)	79/3588 (2.2)	4.27 (2.16-8.46)	<0.001*	0.49 (0.28-0.85)	0.012*
Age ≥ 65 years old (*n* = 3900)						
≥1 days & <30 days	166/780 (21.28)	241/3120 (7.72)	4.17 (3.04-5.71)	<0.001*	0.35 (0.28-0.44)	<0.001*
≥30 days & < 90 days	69/780 (8.85)	155/3120 (4.97)	3.72 (2.48-5.60)	<0.001*	0.39 (0.29-0.52)	<0.001*
≥ 90 days	65/780 (8.33)	161/3120 (5.16)	2.80 (1.80-4.36)	<0.001*	0.53 (0.37-0.75)	<0.001*
Males (*n* = 6275)						
≥1 days & <30 days	259/1255 (20.64)	345/5020 (6.87)	5.26 (4.11-6.73)	<0.001*	0.30 (0.25-0.35)	<0.001*
≥30 days & < 90 days	110/1255 (8.76)	200/5020 (3.98)	5.20 (3.78-7.16)	<0.001*	0.29 (0.23-0.37)	<0.001*
≥ 90 days	71/1255 (5.66)	182/5020 (3.63)	3.46 (2.34-5.12)	<0.001*	0.55 (0.40-0.75)	<0.001*
Females (*n* = 2110)						
≥1 days & <30 days	64/422 (15.17)	129/1688 (7.64)	4.72 (1.99-11.2)	<0.001*	0.26 (0.14-0.46)	<0.001*
≥30 days & < 90 days	19/422 (4.5)	60/1688 (3.55)	4.31 (1.23-15.1)	0.023*	0.17 (0.07-0.40)	<0.001*
≥ 90 days	19/422 (4.5)	58/1688 (3.44)	4.42 (1.26-15.5)	0.02*	0.20 (0.08-0.51)	<0.001*

Low income (*n* = 4235)						
≥1 days & <30 days	175/847 (20.66)	247/3388 (7.29)	4.49 (3.26-6.18)	<0.001*	0.34 (0.27-0.42)	<0.001*
≥30 days & < 90 days	57/847 (6.73)	132/3388 (3.9)	5.22 (3.43-7.93)	<0.001*	0.25 (0.18-0.35)	<0.001*
≥ 90 days	43/847 (5.08)	124/3388 (3.66)	2.90 (1.71-4.93)	<0.001*	0.55 (0.36-0.85)	0.007*
High income (*n* = 4150)						
≥1 days & <30 days	148/830 (17.83)	227/3320 (6.84)	6.06 (4.29-8.57)	<0.001*	0.25 (0.20-0.32)	<0.001*
≥30 days & < 90 days	72/830 (8.67)	128/3320 (3.86)	5.51 (3.52-8.61)	<0.001*	0.31 (0.22-0.43)	<0.001*
≥ 90 days	47/830 (5.66)	116/3320 (3.49)	4.46 (2.66-7.50)	<0.001*	0.44 (0.29-0.67)	<0.001*
Urban (*n* = 3580)						
≥1 days & <30 days	141/716 (19.69)	205/2864 (7.16)	4.34 (3.02-6.23)	<0.001*	0.32 (0.25-0.42)	<0.001*
≥30 days & < 90 days	54/716 (7.54)	107/2864 (3.74)	4.04 (2.45-6.65)	<0.001*	0.38 (0.26-0.55)	<0.001*
≥ 90 days	43/716 (6.01)	98/2864 (3.42)	3.45 (1.98-6.01)	<0.001*	0.53 (0.34-0.82)	0.004*
Rural (*n* = 4805)						
≥1 days & <30 days	182/961 (18.94)	269/3844 (7)	5.89 (4.32-8.03)	<0.001*	0.27 (0.22-0.33)	<0.001*
≥30 days & < 90 days	75/961 (7.8)	153/3844 (3.98)	6.40 (4.34-9.44)	<0.001*	0.22 (0.17-0.30)	<0.001*
≥ 90 days	47/961 (4.89)	142/3844 (3.69)	3.67 (2.23-6.03)	<0.001*	0.49 (0.32-0.73)	<0.001*
CCI scores = 0 (*n* = 5143)						
≥1 days & <30 days	151/850 (17.76)	292/4293 (6.8)	5.57 (3.94-7.88)	<0.001*	0.28 (0.22-0.36)	<0.001*
≥30 days & < 90 days	59/850 (6.94)	137/4293 (3.19)	6.00 (3.78-9.52)	<0.001*	0.26 (0.19-0.36)	<0.001*

≥ 90 days	30/850 (3.53)	105/4293 (2.45)	4.00 (2.14-7.47)	<0.001*	0.44 (0.28-0.70)	<0.001*
CCI scores = 1 (*n* = 1754)						
≥1 days & <30 days	90/453 (19.87)	98/1301 (7.53)	4.08 (2.59-6.42)	<0.001*	0.32 (0.23-0.44)	<0.001*
≥30 days & < 90 days	42/453 (9.27)	58/1301 (4.46)	5.94 (3.50-10.1)	<0.001*	0.24 (0.16-0.38)	<0.001*
≥ 90 days	23/453 (5.08)	65/1301 (5)	3.76 (2.02-7.01)	<0.001*	0.46 (0.25-0.85)	0.013*
CCI scores ≥ 2 (*n* = 1488)						
≥1 days & <30 days	82/374 (21.93)	84/1114 (7.54)	4.90 (3.10-7.74)	<0.001*	0.30 (0.21-0.42)	<0.001*
≥30 days & < 90 days	28/374 (7.49)	65/1114 (5.83)	2.72 (1.40-5.27)	0.003*	0.45 (0.26-0.78)	0.004*
≥ 90 days	37/374 (9.89)	70/1114 (6.28)	1.89 (0.94-3.82)	0.075	0.70 (0.38-1.28)	0.251
Non-GERD history (*n* = 7326)						
≥1 days & <30 days	211/1362 (15.49)	240/5964 (4.02)	5.78 (4.34-7.70)	<0.001*	0.29 (0.24-0.35)	<0.001*
≥30 days & < 90 days	42/1362 (3.08)	112/5964 (1.88)	3.28 (1.89-5.69)	<0.001*	0.40 (0.28-0.58)	<0.001*
≥ 90 days	30/1362 (2.2)	94/5964 (1.58)	2.38 (1.19-4.76)	0.014*	0.54 (0.34-0.86)	0.009*
GERD history (*n* = 1059)						
≥1 days & <30 days	112/315 (35.56)	234/744 (31.45)	2.27 (1.29-4.01)	0.005*	0.48 (0.31-0.76)	0.001*
≥30 days & < 90 days	87/315 (27.62)	148/744 (19.89)	3.34 (1.87-5.96)	<0.001*	0.38 (0.24-0.60)	<0.001*
≥ 90 days	60/315 (19.05)	146/744 (19.62)	2.13 (1.14-3.98)	0.018*	0.69 (0.41-1.14)	0.15
Non-H_2 blocker user (*n* = 3664)						
≥1 days & <30 days	49/432 (11.34)	127/3232 (3.93)	4.01 (2.17-7.42)	<0.001*	0.45 (0.30-0.66)	<0.001*

≥30 days & < 90 days	13/432 (3.01)	61/3232 (1.89)	6.96 (3.33-14.6)	<0.001*	0.21 (0.12-0.35)	<0.001*
≥ 90 days	11/432 (2.55)	70/3232 (2.17)	0.63 (0.09-4.59)	0.647	4.40 (1.15-16.8)	0.031*
H_2 blocker user (*n* = 4721)						
≥1 days & <30 days	274/1245 (22.01)	347/3476 (9.98)	4.31 (3.32-5.59)	<0.001*	0.32 (0.26-0.39)	<0.001*
≥30 days & < 90 days	116/1245 (9.32)	199/3476 (5.72)	4.00 (2.85-5.61)	<0.001*	0.36 (0.27-0.47)	<0.001*
≥ 90 days	79/1245 (6.35)	170/3476 (4.89)	3.38 (2.29-5.00)	<0.001*	0.46 (0.33-0.64)	<0.001*

Abbreviations: CCI, Charlson comorbidity index; GERD, gastro-esophageal reflux disease; OW, overlap weighting; PPI, proton pump inhibitor;

* Logistic regression model, Significance at P < 0.05

† Adjusted for age, sex, income, region of residence, CCI score, H2 blocker dates, and the number of GERD treatment
